# Editorial: Traumatic Brain Injury As a Systems Neuroscience Problem

**DOI:** 10.3389/fnsys.2016.00100

**Published:** 2016-12-09

**Authors:** Han-Chiao I. Chen, John F. Burke, Akiva S. Cohen

**Affiliations:** ^1^Department of Neurosurgery, Perelman School of Medicine, University of PennsylvaniaPhiladelphia, PA, USA; ^2^Philadelphia Veterans Affairs Medical CenterPhiladelphia, PA, USA; ^3^Department of Neurosurgery, University of California, San FranciscoSan Francisco, CA, USA; ^4^Department of Anesthesiology and Critical Care Medicine, Children's Hospital of PhiladelphiaPhiladelphia, PA, USA; ^5^Department of Anesthesiology and Critical Care Medicine, Perelman School of Medicine, University of PennsylvaniaPhiladelphia, PA, USA

**Keywords:** traumatic brain injury, neural circuits, neural networks, systems neuroscience, neuromodulation

Traumatic brain injury (TBI) has gained prominence in the public consciousness as a significant medical problem, especially in light of the recent conflicts in Iraq and Afghanistan and the ongoing discussions of head injuries in sports. Rightfully so, there has been significant energy invested in studying the perturbations of molecular cascades and cellular function in TBI (Mcintosh et al., 1998; Giza and Hovda, 2014; Boychuk et al., 2016) and the relevant neuropathological findings of this condition (Smith et al., 2013). Investigations in these areas have helped guide the clinical treatment of patients during the acute phase of injury and provided a link to long-term outcomes such as chronic traumatic encephalopathy and Alzheimer's disease. However, many clinical symptoms associated with TBI, including cognitive, neuropsychiatric, and consciousness disorders, and issues related to functional recovery from TBI are not easily understood within the frameworks of cellular biology and neuropathology alone. Systems neuroscience examines the activity of neural circuits and how they relate to behavior and function. TBI disrupts neural circuit function, and therefore examining TBI through the lens of systems neuroscience can generate new insights into the deficits experienced by patients and how these problems can be addressed. The objective of this Research Topic in *Frontiers in System Neuroscience* is to present some of the latest findings and views regarding the pathophysiology and treatment of TBI from a systems neuroscience perspective.

While myriad in presentation, many of the ailments that afflict TBI patients beyond the acute phase of the injury can be attributed to the failure of neural circuit systems. The deficits and disorders that mark the subacute and chronic periods of TBI are primary drivers of TBI-associated disability, which affect at least 5.3 million individuals in the United States (Thurman et al., 1999). This disability creates significant burdens for individual patients, their caregivers, and society at large, and it contributes significantly to the $76.5 billion expended on TBI annually (CDC estimate; Injury Prevention and Control: Traumatic Brain Injury and Concussion, 2016). In patients with mild TBI's, upwards of 15% of patients experience persistent symptoms (Marshall et al., 2015), which can include cognitive and memory impairment, neuropsychiatric conditions (e.g., depression and post-traumatic stress disorder), and sleep disorders. With more severe injuries, these problems are accentuated, and other conditions, including movement disorders (Krauss, 2015), disorders of consciousness (Giacino et al., 2014) and post-traumatic epilepsy (Annegers et al., 1998), become more relevant. Outside the context of TBI, the abnormal neural circuitry underlying these various conditions have been the subject of significant study. However, pinpointing the network etiology of specific symptoms after TBI has been difficult because of the heterogeneous nature of the injuries and symptoms across patients. For example, the nature of the memory deficits created by TBI remains unclear, in part because both short-term and episodic memory, which are supported by different neural circuits, are affected. This and other similar discrepancies highlight the need to investigate TBI as a systems neuroscience problem.

Given the above considerations, we believe that establishing the mechanisms by which traumatic disruption of brain networks induces deficits will require a multi-modal approach that cuts across disciplines. The first section of this Research Topic is comprised of three papers that offer different governing principles for understanding how TBI impacts neural circuit function. Bigler et al. describes how quantitative image analysis can be used to correlate changes in brain structure and connectivity to neuropsychological outcomes. Wolf and Koch posit that post-TBI deficits are due to disruptions in the timing of neuronal communication as a result of axonal injury. Carron et al. suggest that the symptomology of TBI can be viewed as aberrations of sensory system processing and that changes in cortical interneuron activity likely explain the hyperexcitability and alterations in neuronal encoding seen after TBI. These papers provide insight into how TBI perturbs brain network function and will, we hope, serve as guides for future investigations in this area.

Although the focus of this Research Topic is on the relationship between TBI and brain networks, network activity is ultimately built upon the function of individual neurons. As such, the second section of this Topic includes four papers that describe the effect of altered cellular metabolism and mitochondrial function on neural activity. Sun and Jacobs demonstrates how targeting cyclophilin-D and its effects on mitochondrial permeability transition pore opening could reverse TBI-induced abnormalities of intrinsic neuronal firing properties and reduce synaptic hyperexcitability. Continuing on the theme of mitochondrial dysfunction, Fischer et al. describe the correlation between TBI and increased mitochondrial fission, which may impair the survival of newborn neurons in the hippocampus. Wilson et al. explore how increased levels of phosphodiesterase isoforms may contribute to impaired hippocampal synaptic plasticity. Finally, Dash et al. show a decreased level of methionine and its metabolites in patients with severe TBI, which could lead to altered epigenetic regulation. These studies point to the many different cellular mechanisms that can contribute to neuronal, and thus neural circuit, dysfunction after TBI.

One of the most exciting aspects of studying TBI from a systems neuroscience perspective is the possibility of developing novel therapies specifically for circuit dysfunction. The third and final section of this Research Topic contains four articles that examine therapeutic modalities based on how they affect brain network function. Pevzner et al. illustrate how the oscillatory activity of the brain is altered after TBI and how low-frequency stimulation of the medial septum could restore normal oscillatory rhythms in cognitive circuits. Girgis et al. survey the biochemical and circuitry changes in the injured hippocampus and review other potential stimulation targets. The article by Murugan et al. documents the effects of the flavonol compound kaempferol on reversing large-scale deficits in neural activity as measured by cerebral blood flow. Lastly, Butler et al. studied how inhibiting the mTOR pathway limited post-traumatic hippocampal neurogenesis and mossy fiber sprouting, a potential mechanism for suppressing post-traumatic epileptogenesis. These articles raise the possibility of a new generation of more effective interventions for TBI.

One of the primary reasons that TBI continues to have a widespread and devastating impact on patients is that there are few options for treating the long-term sequelae of this condition. Brain network activity is the closest biological correlate to clinical function, and thus it makes sense to study neural circuits and their dysfunction after TBI as a means of identifying new therapeutic targets. In this Research Topic, we have highlighted several different approaches for examining TBI-induced changes in the function of neural circuits and individual neurons. Future studies should continue this trend of studying network dysfunction after TBI and linking changes in neuronal metabolism and gene expression to neural circuit activity. Special attention will need to be paid to understanding how heterogeneous injuries can lead to common symptoms. We believe that this systems neuroscience approach will promote new interpretations of TBI, which will lead to novel therapeutic interventions, such as those presented in this Topic, and improved clinical outcomes for patients.

## Author contributions

All 3 authors were co-editors on the Research Topic entitled, “Traumatic Brain Injury As a Systems Neuroscience Problem.”

## Funding

This work was supported by the National Institutes of Health (R37HD059288 to AC) and the Department of Veterans Affairs (IK2-RX002013 to HC).

### Conflict of interest statement

The authors declare that the research was conducted in the absence of any commercial or financial relationships that could be construed as a potential conflict of interest.
